# ‘Let’s Move It’ – a school-based multilevel intervention to increase physical activity and reduce sedentary behaviour among older adolescents in vocational secondary schools: a study protocol for a cluster-randomised trial

**DOI:** 10.1186/s12889-016-3094-x

**Published:** 2016-05-27

**Authors:** Nelli Hankonen, Matti T. J. Heino, Vera Araujo-Soares, Falko F. Sniehotta, Reijo Sund, Tommi Vasankari, Pilvikki Absetz, Katja Borodulin, Antti Uutela, Taru Lintunen, Ari Haukkala

**Affiliations:** School of Social Sciences and Humanities, University of Tampere, Kalevankatu 4, 33014 Tampere, Finland; Faculty of Social Sciences, University of Helsinki, Helsinki, Finland; University of Newcastle Upon Tyne, Newcastle Upon Tyne, UK; UKK Institute, Tampere, Finland; School of Health Sciences, University of Tampere, Tampere, Finland; National Institute for Health and Welfare, Helsinki, Finland; Faculty of Sport and Health Sciences, University of Jyväskylä, Jyväskylä, Finland

**Keywords:** Physical activity, Sedentary behavior, Adolescents, School-based intervention, Vocational school, Accelerometer, Behaviour change, Complex intervention

## Abstract

**Background:**

Physical activity (PA) has been shown to decline during adolescence, and those with lower education have lower levels of activity already at this age, calling for targeted efforts for them. No previous study has demonstrated lasting effects of school-based PA interventions among older adolescents. Furthermore, these interventions have rarely targeted sedentary behaviour (SB) despite its relevance to health. The Let’s Move It trial aims to evaluate the effectiveness and the cost-effectiveness of a school-based, multi-level intervention, on PA and SB, among vocational school students. We hypothesise that the intervention is effective in increasing moderate-to-vigorous-intensity physical activity (MVPA), particularly among those with low or moderate baseline levels, and decreasing SB among all students.

**Methods:**

The design is a cluster-randomised parallel group trial with an internal pilot study. The trial is conducted in six vocational schools in the Helsinki Metropolitan area, Finland. The intervention is carried out in 30 intervention classes, and 27 control classes retain the standard curriculum. The randomisation occurs at school-level to avoid contamination and to aid delivery.

Three of the six schools, randomly allocated, receive the ‘Let’s Move It’ intervention which consists of 1) group sessions and poster campaign targeting students’ autonomous PA motivation and self-regulation skills, 2) sitting reduction in classrooms via alterations in choice architecture and teacher behaviour, and 3) enhancement of PA opportunities in school, home and community environments. At baseline, student participants are blind to group allocation. The trial is carried out in six batches in 2015–2017, with main measurements at pre-intervention baseline, and 2-month and 14-month follow-ups. Primary outcomes are for PA, MVPA measured by accelerometry and self-report, and for SB, sedentary time and breaks in sedentary time (accelerometry).

Key secondary outcomes include measured body composition, self-reported well-being, and psychological variables. Process variables include measures of psychosocial determinants of PA (e.g. autonomous motivation) and use of behaviour change techniques. Process evaluation also includes qualitative interviews. Intervention fidelity is monitored.

**Discussion:**

The study will establish whether the Let’s Move It intervention is effective in increasing PA and reducing SB in vocational school students, and identify key processes explaining the results.

**Trial registration:**

ISRCTN10979479. Registered: 31.12.2015

**Electronic supplementary material:**

The online version of this article (doi:10.1186/s12889-016-3094-x) contains supplementary material, which is available to authorized users.

## Background

Despite widely acknowledged benefits of physical activity (PA), adolescents worldwide engage in far less PA than is recommended [1]. In adolescence, PA tends to decline with age and vary by gender, with boys reporting higher PA levels than girls [2]. Increasing evidence points to detrimental effects of excessive sedentary behaviour (SB) even among those with sufficient levels of PA [3]. Socioeconomic status (SES) is one of the central correlates of life expectancy, health and health behaviour, and is also related to PA and SB in adolescents, with lower SES youth engaging in less PA and in more SB [4].

Schools are a favourable setting for interventions aimed at adolescents: they reach a majority of potential participants, who also spend a major part of their time there. Although schools are a very promising setting for PA promotion [5], variance in PA intervention effect sizes, so far, is high [6], and interventions tend to have mainly short-term effects [7] and change PA especially among girls [8].

With prior studies focusing on children, school-based PA interventions among older adolescents or in vocational schools are rare [5]: Our systematic review [9] showed that only 10 RCTs of school-based interventions to improve PA or SB among 15–19 year-olds have thus far been reported, and none of the trials had high methodological quality. Only a few of existing trials have assessed long-term effects, and of those who have, none were effective [9]. An analysis of the behaviour change techniques (BCT) and other intervention features characterising effective and ineffective trials suggested the potential of including a larger number of BCTs and certain BCTs (e.g. information about social and environmental consequences, self-monitoring of PA) [9], in line with other evidence (e.g., [10]).

Effective school-based PA and health promotion programs have been designed using behavioural theories and intervene at several levels (e.g. not classroom-based education only) (e.g. [11–13]). The possible trade-off between effectiveness and resources should be assessed with cost-effectiveness analyses. Generally, school-based PA programs have medium costs and medium-sized effects [14], but evidence among older adolescents is scarce. With complex interventions, comprehensive process evaluation has the potential to identify causal mechanisms of change and components critical to success [15]. However, theoretical determinants identified as prospective predictors of youth PA (e.g. [16, 17]) have rarely been studied as mediators in school-based interventions in this age group.

Self-report assessments of PA and especially SB contain some uncertainty as well as high variation in individual reporting, and accelerometry is a state-of-the art method to reliably assess activity [18]. As accelerometry does not capture all types of PA (e.g., water sports), self-reported PA could complement but not replace objective assessment. In previous trials, objective assessment of outcomes beyond self-report has been used in only three small trials (sample sizes of 94 or lower), using individual-level analyses only despite nestedness in classes [9].

Thus, large gaps exist in the evidence concerning effectiveness of interventions to improve activity behaviours among lower educated youth. Answering to the need for high-quality school-based PA intervention studies for older adolescents, this study aims to fill gaps in current evidence by including objective measures of behaviour and body composition, avoiding an underpowered study design, taking into account clustering in classes, and following participants up to 12 months after the end of the intensive intervention (14-month follow-up).

**Fig. 1:**
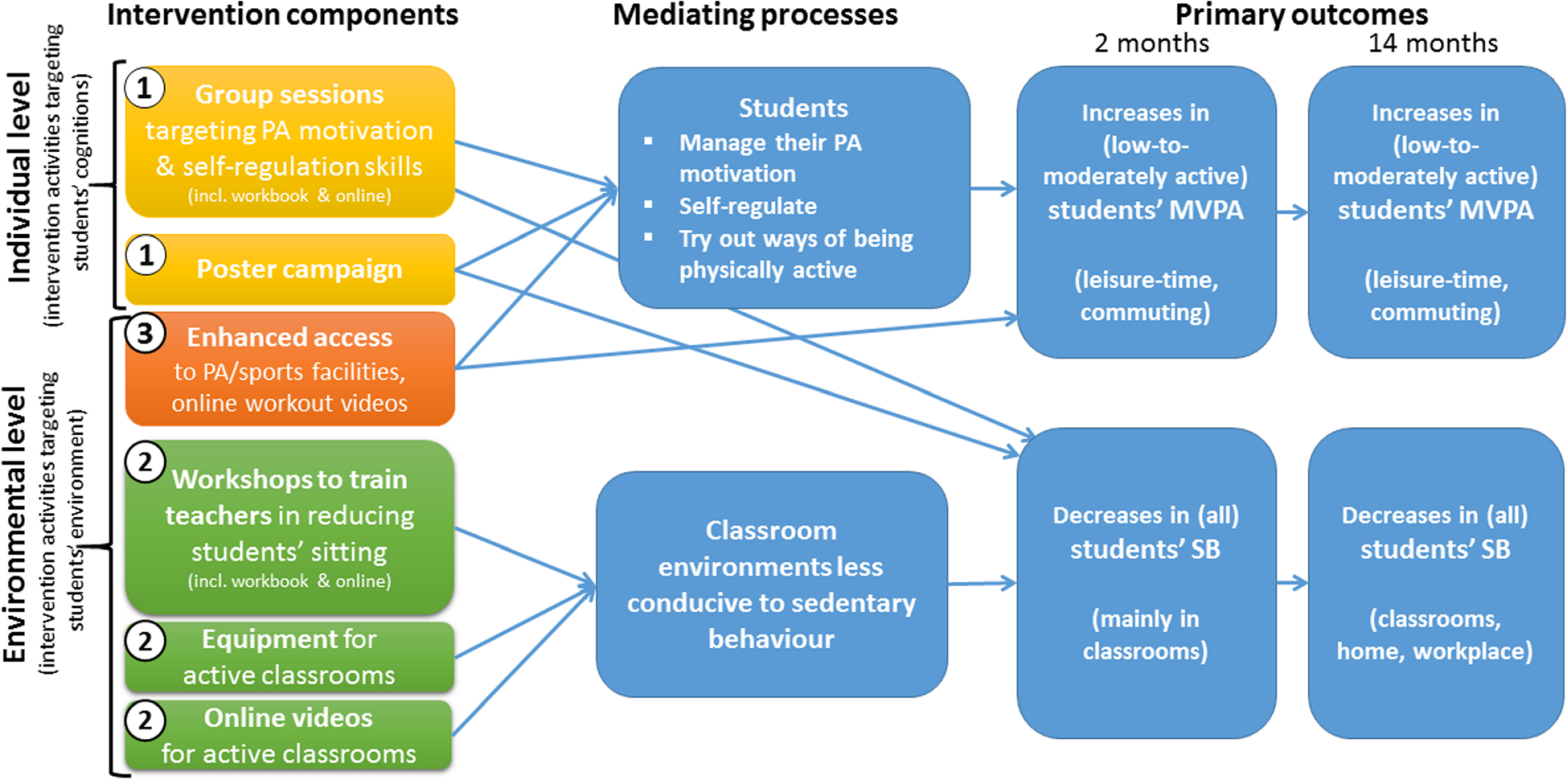
Simplified logic model linking intervention components to hypothesized mediating processes and primary outcomes

The ‘Let’s Move It’ (Fig. 1) is a complex theory- and evidence-based intervention for vocational schools that includes elements aiming at increasing individuals’ autonomous motivation for PA, self-regulation skills for initiating and maintaining regular PA, and at enhancing environmental opportunities for reducing SB and increasing PA. The intervention components targeting PA were designed especially for and with adolescents with low or moderate levels of PA [19]. Our randomised controlled feasibility study [20] in 2014 demonstrated the acceptability of the Let’s Move It curriculum for both teachers and students, and identified further improvement needs, which led to an optimised version of the intervention to be tested in the current cluster-randomised trial.

The objective of this paper is to describe the study protocol for a cluster RCT designed to evaluate the effectiveness and cost-effectiveness as well as processes of the Let’s Move It programme compared with usual curriculum for students in vocational schools. It builds on the study protocol developed in our feasibility trial [20].

## Methods/Design

### Aims of the study

The primary aim of the trial is to determine the effectiveness of the Let’s Move It intervention on changes in students’ physical activity and sedentary behaviour over two months (post-intervention, T3) and 14 months (long-term follow-up, T4), specifically in the following primary outcome measures:

1. Moderate-to-vigorous intensity PA (MVPA), as measured by accelerometry and self-report
2. Sedentary behaviour: a) Sedentary time, and b) Breaks in sedentary time (accelerometry)

We hypothesise that the intervention is effective in increasing or maintaining PA and reducing SB, compared to control group, among girls and boys, both immediately and one year after the intensive intervention (i.e., 2 and 14 months from baseline).

As the trial is designed especially for adolescents with low PA at baseline, we expect to detect a significant difference in change in MVPA among students with low or moderate levels of MVPA at baseline. We also aim to examine the cost-effectiveness of the intervention, in terms of determining cost of incremental activity. Additionally, we will evaluate changes in secondary outcomes among students (e. g. other indicators of activity, body composition, physical and mental well-being, as well as psychosocial variables) and teachers. Process evaluation will be conducted to examine mechanisms of change, implementation fidelity and key components of the intervention, as well as responses and effectiveness in subgroups.

### Trial design

The design is a pragmatic, cluster randomised controlled parallel group trial with one intervention arm (Let’s Move It programme) compared with control arm (standard curriculum). The trial is a superiority trial. The first and second batches of the trial serve as an internal pilot study. The internal pilot data was subject to interim analysis in line with the analysis plan to investigate previous assumptions and to make adjustments to the data collection plan.

Control and intervention arms include altogether 57 classes (intervention *k* = 30, control *k* = 27). The school is the unit of cluster randomisation, but classes are the units of cluster analysis. A cluster randomisation based on school was chosen for practical reasons and to prevent contamination. The level of class (student group) will be the cluster unit of analysis, as the essential part of the intervention is delivered in the group setting, and the nestedness of individuals in the classes may influence the individual outcomes due to e. g. group dynamics and emerging norms. Matching schools by type of educational tracks they offer (e.g. business, nursing) [21] results in the same educational tracks being allocated to both intervention and controls. Paired units were randomly assigned to intervention or control. The intervention is provided as normal part of the school. The details of the trial have been planned to avoid the typical sources of bias in health behaviour RCTs [22], including using a strict protocol for participant contact (performance bias), cluster randomization (contamination bias), and objective outcome measurement.

### Study setting

Vocational schools. The study will be conducted in the Helsinki Metropolitan area, Finland. The list of study sites can be obtained from the first author.

### Participants and eligibility criteria

Participants are vocational students in study year 1 and 2 (typically aged 15–17). All students will be invited to take part. Of educational tracks, those that meet the following criteria were selected for the study: Those educational tracks whose 1) students, compared to other educational tracks in the Finnish National School Survey, report on average higher levels of SBs and 2) report lower levels of PA on their leisure-time, 3) student intake in the national system is high to meet sample size requirements, and 4) future occupations that are either sedentary or require adequate/good physical condition. These educational tracks were selected based on evidence (Finnish National School Health Promotion survey, THL) and active consultation with the study scientific steering committee and with stakeholder expert group meetings, and the educational tracks were ranked as follows: 1. Business and administration, 2. Business information technology, 3. Nursing, 4. Hotel, restaurant and catering studies, 5. Tourism, 6. Car mechanics. The final selected combination of tracks (1.-4.) allowed for an even proportion of boys and girls in the trial. Next, the eligibility criteria are explained for schools, classes, individual students, and individual teachers.

(1) **Vocational schools**. Of the vocational schools within the study region, we decided to select schools that have the highest number of starting students in selected educational tracks (to meet sample size requirements). Six school units were recruited. In these schools, the trial aims to include the maximum number of classes in years 1 and 2 in the selected educational track present in the school during the study period (and not in vocational work practice outside school).

Schools had to agree to randomisation, allow teachers to participate in the Let’s Move It workshop training, scheduling the Let’s Move It student program in their curriculum and be representative of Finnish vocational schools.

(2) **Classes**. Inclusion criteria include 1) classes of vocational students in the study year 1 or 2; and 2) possibility to integrate 6 sessions of Let’s Move It into the standard curriculum (e.g. either within the Health Education course or another alternative course catered in the first study period after baseline measurement). The exclusion criteria are 1) classes catering for students with severe physical and mental disabilities, 2) classes for students with insufficient knowledge of the Finnish language to take part in group interventions and use written materials, e.g., preparatory classes for immigrants, and 3) classes that attend lessons in vocational schools only 1 day per week or less (due to partial on-the-job learning or high school teaching).

(3) **Students**. Inclusion criteria for students are being listed as students in the class and/or attending the class’s lessons. Exclusion criteria include physical condition hindering taking part in bioimpedance measurement (e. g. heart device).

(4) **Teachers**. Inclusion criteria for teachers include being core class or vocational class teachers whose teaching involves a large amount of sitting or static/burdening work positions and who teach the included classes in the study period.

### Recruitment

Recruitment of schools, classes and students occurs through several phases.

(1) Six vocational schools or school units were recruited in autumn 2014. The schools in the Helsinki Metropolitan area that cater for the selected educational tracks were ranked in the order of size of intake of students in that educational track. The suitability according to selection criteria determined the order in which the eligible upper secondary schools were approached to participate. Once randomised, schools either deliver Let’s Move It as part of their curriculum (intervention) or continue to offer PE and health education as part of their usual curriculum (control).

A letter was sent to directors of the selected schools (summer/autumn 2014), informing about the study and inviting participation, including a letter of support from the Finnish Board of Education recommending participation in the Let’s Move It trial. Then, the principal was contacted by phone by a member of the research team, following by a face-to-face meeting to outline the requirements of the study. Invitations to participate were sent to schools, and if a selected school declined, a letter was sent to the next eligible school on the list that matches the matched-pair school according to the criteria. School principals gave consent prior to randomization and thus were blind to randomisation. Out of all school units (8) contacted, two schools did not accept the request for the research team to present the project more in detail, due to heavy workload or other similar interventions going on already at the school. Six schools consented.

(2) Following randomisation of schools, eligible classes of first and second year students were enrolled, typically with final confirmation a few weeks before the baseline measurement. The trial was commenced in January 2015, and data are collected in six batches, with the same educational tracks measured in parallel in both intervention and control arms: business/IT (Batches 1 and 2, spring 2015), nursing (Batches 3 and 4, autumn 2015), and hotel, restaurant and catering (Batches 5 and 6, spring 2016, ongoing) (Fig. 3).

(3) For each of the included classes, researchers give an oral presentation about the study and provide written study information and consent forms. All students are asked to assent to data collection, but they are also informed about the voluntary nature of the study. The students in the participating classes are provided a letter outlining the study and a consent form. According to Finnish law, in research on 15-year-old youth and older, their own consent to participate in the study data collection is sufficient, and guardians only need to be informed about the study. The research assistants collect the signed consent forms. The Let’s Move It intervention is offered as part of the normal school curriculum, so all students normally participate in the intervention activities, regardless of whether they have given consent for study measurements.

(4) In the staff meeting, teachers are given a short oral presentation about the study and the teacher workshops (intervention arm). They are then sent an online questionnaire. Part of the agreement with the participating schools means that teachers are permitted to fill in the assessment questionnaires and attend the training workshops during work time. Participation is voluntary.

### Interventions

#### Active arm: intervention overview

The intervention objective is to increase total PA, more specifically: 1) increase moderate-to-vigorous intensity PA (MVPA), especially among those with low levels of PA, 2) decrease time spent in SBs, and 3) increase the number of breaks in SB. The intervention design was preceded by a comprehensive needs assessment and preliminary research among the target group [19] as well as by synthesis of prior evidence [9] and a randomised acceptability and feasibility (rehearsal) trial [20]. The program was designed specifically for those with low or moderate baseline PA levels: The student panel for the initial development of programme purposefully consisted of youth reporting low levels of PA, and after the feasibility trial, the feedback for optimization was collected among participants excluding those with already high levels of PA. Nevertheless, Let’s Move It intervention is delivered as a universal intervention for all students, in order to minimise the risks of inequalities in accessing the intervention and any potential for stigma in case the intervention would only be targeting a subgroup within the school. The design process of the Let’s Move It intervention made sure that this intervention could fit into the school curriculum. The intervention is based on theoretical and empirical insights from behavioural science: e.g. self-determination theory [23], especially its goal content and organismic integration theories, self-regulation and planning theories [24, 25], and habit formation [26], taking into account group motivational interviewing principles for the delivery of the actual intervention activities and active ingredients [27–29].

The intervention logic model for changing student PA is different from that of changing student SB (Fig. 1), partially due to the differing nature of and perceived familiarity with the behaviours. In this intervention study, PA behaviours are hypothesised to change via a set of conscious motivational and self-regulation processes. These are targeted in group sessions using pair and group discussions. In this group setting, graded, step-wise self-guided experimentation and prompting practice with PA is used, encouraging students to select their own leisure time PA/sports type and setting. This approach is used to increase behaviour maintenance: Regularly incorporating PA in daily life may be more likely to result in maintenance across time, even outside of school terms, compared to an intervention providing PA within the sessions in schools.

Changes in SB were designed to, primarily, be introduced by environment: changes in the physical choice architecture in classrooms (e.g. gym balls as chairs) and teacher-led changes (e.g. increasing breaks in sitting in class). In the Let’s Move It student sessions, consequences of excessive SB and tips for reducing SB in one’s daily life are briefly introduced. The parallel poster campaign aims at reinforcing these cognitive and attitudinal changes. All this goes in tandem with personal experiences of less sitting in the classrooms. The latter is hypothesised to mainly lead to changes in intentions and attitudes, supported by the educational elements of sessions and posters. The intervention encourages the students to generalise the newly adopted SB restriction behaviour in the leisure (e.g. home) and workplace contexts. Finally, the intervention designers acknowledge the overlapping and intertwining nature of these behaviours: The activities encouraged in the student group sessions include not only MVPA but also light PA, substituting sedentary activities (encouraging even small improvements).

#### Basic intervention components

The program contains 1) six intracurricular group sessions, and a later booster session, as well as supporting online and poster materials throughout the study, 2) teacher-led activity breaks and other SB reduction practices in classrooms, and 3) increase of other environmental opportunities for PA (e. g., lower-cost participation in neighbourhood sports centres) (Fig. 1). More specific description of each component follows:

1. ***The PA group intervention*** consists of six interactive face-to-face *group sessions*, ranging from 45-minutes to 60 min each, which have been designed to target student PA motivation and self-regulation skills. Trained facilitators (part of the research team) deliver these group sessions as part of health education or a similar course included in the school curriculum. BCTs support improvement of both the amount and the quality of motivation, and the progress from motivation to goal setting and further self-regulation. The intervention also aimed to target dysfunctional beliefs regarding PA by e.g. emphasising well-being motive over weight loss motive, and benefits of even small increases in activity. Table 1 lists key interaction principles and Table 2 the key BCTs as defined by the BCT Taxonomy v1 [30]. *The website and workbook* contain additional materials for students. To support acquisitions during the sessions as well as retention, the key messages of the intervention are portrayed in a sequentially proceeding *poster campaign* in school. The posters were designed together with an advertisement agency and are based on the results obtained during the needs assessment phase and aligned with behavioural science and BCTs (targeting specific theory-based mechanisms). In addition to wall posters, school canteens are provided with table triangle stands reiterating the most important messages, and providing examples and guidance for self-enacting the BCTs taught and used in the intervention sessions and the workbook.

**Table 1:** Interaction principles for intervention providers (adapted based on Deci & Ryan, [23]; Miller & Rollnick, [29])

1. Show empathy for students	
2. Ask open questions	
3. Roll with students’ resistance	
4. Evoke change talk	
5. Show interest in students’ experience and perspectives	
6. Provide students with options and choices	
7. Provide students with structure and agenda	
8. Use reflective listening	
9. Validate students’ concerns	
10. Provide positive feedback

**Table 2:** Some of the most important behaviour change techniques delivered in the Let’s Move It student group intervention’s core sessions. Full list of BCTs is reported by session and by exercise in a separate manuscript. Based on BCT Taxonomy v1 [30]

Key behaviour change techniques		1	2	3	4	5	6
2.3	Self-monitoring of behaviour	x	x	x	x	x	x
5.1	Information about health consequences	x	x	x	x	x	x
5.6	Information about emotional consequences	x	x	x		x	x
5.3	Information about social and environmental consequences	x	x	x	x	x	x
5.2	Salience of consequences	x				x	x
5.4	Monitoring of emotional consequences	x	x	x	x		
13.5	Identity associated with changed behaviour	x			x	x	x
1.1	Goal setting (behaviour)		x	x	x	x	x
8.7	Graded tasks		x		x	x	x
1.4	Action planning		x	x	x	x	x
4.4	Behavioral experiments		x	x			
3.1	Social support		x	x	x		
2.2	Feedback on behavior		x	x	x	x	x
1.2	Problem solving (coping planning)		x	x	x	x	x
7.1, 12.1	Prompts/cues, Restructuring the physical environment		x	x			
1.6	Discrepancy between current behaviour and goals			x	x	x	x
1.5	Review behavior goals			x	x	x	x
8.1	Behavioural practice			x	x		
4.1	Instruction on how to perform a behaviour			x	x		
6.1	Demonstration of behaviour			x	x		
4.2	Information about antecedents of behaviour				x	x	x
2. ***The classroom SB reduction program***, delivered by teachers, involves decreasing students’ sedentary time and introducing breaks in sedentary time in the classroom by altering choice architecture providing equipment for light-intensity activity, gym balls to replace part of the classroom chairs and standing desks to replace part of the classroom tables, and by the use of active teaching methods and the introduction of activity breaks during class. The teachers are trained in* three 90-minute workshops*, and the training aims at creating sustained habitual behaviours in providing SB reduction for students. Support materials for teachers include: a) a 62-page *booklet* demonstrating a range of strategies to reduce student sitting, with BCTs to support the incorporation of sitting reduction as a regular routine (habit formation); b) *online materials* providing strategies and BCTs as well as video-led sitting reduction or activity breaks for classrooms, and useful external links, and c) *posters* demonstrating various forms of activity breaks with explicit links to subsequent benefits (e.g., vigour, increased muscle strength, relaxation).
3. ***Enhanced access to PA opportunities*** include a) maximising access to existing *school PA facilities*, b) establishing *partnerships* with community sporting groups and/or organisations to provide salient opportunities for students as well as reduced price deals (for leisure-time PA), and c) providing students with *online exercise videos* to guide home-based workout. These videos were tailored to youth with little experience in PA and low fitness (e.g., typical length 10 min), and required no gear. There are six videos that include workout sessions targeting muscle strength, flexibility, and aerobic fitness, ranging between 9 and 20 min in length. These videos were designed by a certified personal trainer. A young male professional personal trainer demonstrates the workout, with a female voice guiding through the activities (prompt practice, provide instruction on how to perform the behaviour, demonstration of the behaviour) using a positive, encouraging approach in line with the ethos of the Let’s Move It. Students were encouraged to combine multiple videos to construct their own session based on their own preference and to use their own music, in order to promote experience of autonomy.

#### Maintenance/boosters across the three components

Specific elements were designed to support maintenance of the changed behaviours, listed below under each of the components. For each intervention school, a *Let’s Move It school team* is founded, composed of voluntary teachers and the Let’s Move It project coordinator as their contact person. It function is to cover all these elements and aim at increasing generalisability, sustainability, and maintenance of the Let’s Move It intervention.

1. One *booster session* is delivered over the following 3-6 months to support maintenance of the changes. Students are invited to follow Let’s Move It *social media* channels with regular “boosters” (e.g., pictures of Let’s Move It posters, suggestions for seasonal PA in the area). *Posters* and table triangle stands are still visible in schools after the intensive intervention until the end of the trial.
2. The third *workshop* for the teachers is scheduled after the intensive 2-month intervention period. Two quarterly *email newsletters* are sent to teachers to remind of the importance of and strategies for sitting reduction. Additionally, school heads/educational track directors are requested to discuss the Let’s Move It activities in *teacher meetings as part of the agenda* (slideshow materials and discussion questions are provided to them). A teacher tailored *year-long calendar poster* is placed at the wall of the teachers’ staff office to remind of the various sitting reduction activities teachers selected in the workshops to use throughout the year. Enthusiastic teachers and other staff members are invited to affiliate with the school team that enhances maintenance of intervention activities in schools after the 2-month intensive intervention (e.g., recycling posters, raising the question of sitting reduction in staff meetings).
3. The school team is requested to ensure continued high access to sports facilities. The use of online videos is promoted in the face-to-face booster session and posters.

A central element across all intervention is autonomy supportive interaction designed to foster self-determined motivation in both students and teachers, aiming for sustained motivation and behaviour (Table 1). Active learning techniques and gradual, step-wise progress, with a celebration of even small changes is emphasised. Sample posters and other materials are shown in the Additional file 1. Intervention components and their development are described more fully elsewhere [19].

It should be noted that the intervention pertains to both the class cluster level (e.g. Let’s Move It student sessions, teacher-led activity breaks in classes) and individual level (e.g. tailored goals and action plans), as well as school cluster level (poster campaign, teachers’ workshop & PA equipment, school team). All students of the included classes participate in the Let’s Move It sessions as part of their normal curriculum, i.e., the voluntariness involves participation in the research measurements.

#### Control condition

Control group students receive teaching as usual, i.e., standard curriculum. Whereas the feasibility trial control group received leaflets informing of consequences of PA and SB, the main trial uses standard school curriculum as the comparison, because 1) comparison to standard only provides more relevant information and 2) the leaflets were perceived unfeasible [20].

#### Strategies for enhancing and monitoring intervention fidelity

The intervention sessions are carefully manualised. All intervention facilitators are carefully trained, with role-play exercises and ongoing revisions. Intervention facilitators keep track of components delivered, as well as the quality of delivery (e.g., interaction elements), by filling in a self-assessment form after each session, to assess whether the intervention was delivered as intended and to ensure high fidelity. We evaluate the extent to which opportunities to access community and school PA facilities were enhanced, and the fidelity of poster campaign uptake and maintenance. We assess receipt and use of intervention materials (e.g., use of workout and activity break videos) and enactment of the BCTs taught in intervention classes [31].

### Primary outcomes

In line with intervention targets, primary outcomes are, for PA, changes in MVPA and for SB, changes in overall sedentary time and breaks in sedentary time. These are designed to capture key aspects of the behaviours. The objectively measured PA and SB outcomes are based on accelerometry [32]. Self-reported MVPA outcome is needed to measure types of PA that accelerometry does not (e.g., water sports, contact sports not allowing for any attachments to body).

### Secondary outcomes and process evaluation

Secondary outcomes among students include objectively measured body composition (muscle and fat mass), other indicators of activity (e.g. self-reported SB), physical and mental well-being, and psychosocial variables (e.g. behavioural automaticity). Key secondary outcomes among teacher participants include self-reported sitting reduction activities and observed student behaviour.

In order to examine mechanisms of change and implementation fidelity, processes will be evaluated using both quantitative and qualitative methods. Evaluated process variables include hypothesised mediators, including psychological theoretical determinants, BCT use, observed teacher sitting reduction behaviours. Intervention fidelity, responses to intervention and effectiveness in subgroups will also be investigated.

### Sample size

The internal pilot study data (the first and second batches) were subject to interim analysis in line with the analysis plan to investigate whether the assumptions for the initial power calculations were correct (e. g., intra-cluster correlation and standard deviations for changes in outcomes). This was done in order to make adjustments to the data collection plan, to avoid collecting too much or too few data. Additionally, accumulation of accelerometry data was investigated. Altogether 502 participants (96 % of total potential) consented to the measurements. Three hundred seventy six participants were delivered the accelerometer, and of these, 267 (71 %) wore the accelerometer device over 10 h of data on at least four days. Descriptive data from the internal pilot study is presented in Additional file 2: Table S1.

We conducted power analyses for all primary outcome measures. For individual-level design, a sufficient number is 176 persons per arm to determine a change of effect size d = 0.30 (e.g. a mean of 8.24 min difference in daily MVPA or 33.44 min difference in daily sedentary time) with 80 % power and an alpha of 5 %, translating to a post-dropout sample size of 352.

Individuals in this trial are nested within clusters (classes) when the intervention is delivered, which may affect the results. Therefore we also calculated the effect of clustering on the power, with a method [33] that took into account the intracluster correlation coefficient (ICC), varying cluster sizes and the expected effect. Data from the internal pilot was used to estimate the ICC and assess the likely mean and SD of cluster sizes. The ICC for MVPA in the internal pilot study varied from 0.033 (self-report measure) to 0.059 (objective measure) and we used a mean cluster size of 17 (SD = 3.83). This resulted in design effects of 1.56 and 2.00, giving us 80 % power to detect d = 0.3 with a sample sizes of 549 and 703, for self-report and objective MVPA measures, respectively. Using the same cluster parameters for the SB measures, with ICC of 0.012 for interruptions to sitting and 0.057 for sedentary time, design effects of 1.21 and 1.96 point to requirements of 425 and 691 students, respectively.

Drop-out rates in previous similar trials [9] with sample totalling more than 100 and containing both genders have varied widely, from 3.1 to 54.1 %. Our internal pilot study showed a dropout of ~25 % from T1 to T3. Due to improvements in follow-up data collection procedures, dropout rates are expected to diminish in batches 3–6. In conclusion, with an expected sample size of ca. 1100 students, and with a dropout of 20 % from T1 to T3 and a further 20 % from T3 to T4, we would be well-equipped to account for clustering in all primary outcome measures with the post-dropout sample size of 704. Assuming 20 % of participants are classified as high-active and therefore excluded from the MVPA analysis, we expect to achieve 93 % power to detect d = 0.3 with individual-level analyses. Due to the drop in SD, this effect translates to an absolute difference of 7.25 min for the target group, instead of 8.24 min for the whole sample. The ICC estimated from the internal pilot study for the low- to moderate-active students’ MVPA change was 0.038, leading to a design effect of 1.65 and subsequently a post-dropout sample size of 580 to reach 80 % power.

### Randomisation procedure and blinding

Prior to starting the trial in 2014, participating schools were randomly allocated to intervention and control arms, using computer-generated random numbers, by a statistician (RS) who was not involved in contacting schools, design of the intervention program, or carrying out the intervention. Because schools differed in educational track and size (large/small), the trial arms were first balanced by matching consenting school units as pairs so that large enough sample size could be ensured for each arm. This was possible as all school clusters were recruited prior to allocation and prevents the problem that heavily uneven-sized trial arms are known to cause [33]. Randomisation was then conducted at the school level and the matched school units (and educational tracks) conveniently fell in intervention or control schools in a balanced way.

On receiving invitation to the study, student participants are blind to randomisation to avoid participation bias. Research staff are not blind to allocation due to impracticalities identified in the feasibility study [20], however, the research procedures and scripts are highly standardised and research staff are carefully trained in order to treat all student participants similarly in both arms. Also, researchers collecting follow-up data are not blind to allocation: as there are major changes present in the intervention schools (e.g., standing desks, poster campaign) and removing these prior to follow-up measurements for the sake of securing outcome assessment blinding would not be justified or feasible. Also, school is the most optimal location to contact the students, so moving measurements to other locations would also not be feasible. However, the contacts and interactions with the participants are carefully scripted in order to provide identical contacts with control and intervention participants and hence not to bias the results. The research assistants are trained over several occasions to adhere to these procedures, and those collecting follow-up measurements were not delivering the intervention to avoid any social desirability effect.

The school principal was contacted by the study team to inform of random allocation. This is necessary as the principal and other staff have to negotiate placing of intervention materials, changes in physical environment, etc. The staff are informed about whether they are intervention or control schools but they are requested not to inform the students prior to baseline, but instead leave all communication regarding the study to the research group.

### Materials and procedures

**Fig. 2:**
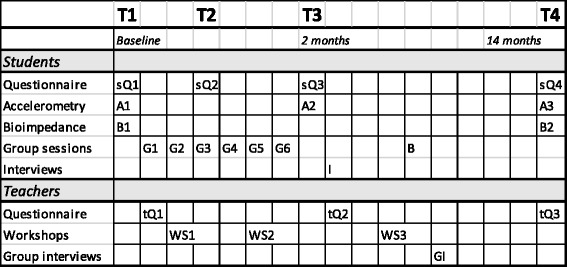
Schedule of interventions and assessments. Students: sQ1-SQ4 = Questionnaires, A1-A3 = Accelerometry measurements, G1-G6 = Group intervention sessions, B = Booster session, B1-B2 = Bioimpedance measurement, I = Interviews. Teachers: tQ1-tQ3 = Questionnaires, WS1-WS3 = Workshops for teachers

**Fig. 3:**
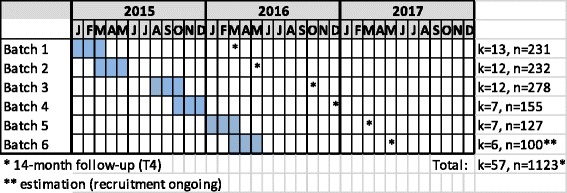
Timing of data collection and (expected) sample size in each batch (*k* = student groups, *n* = individual students)

Through the conduct of our acceptability and feasibility trial [20], we developed and tested many of the instruments and data collection methods that are used in the trial described in this protocol. Data is collected from students at four time points: pre-intervention baseline (Time 1, T1), during intervention after the 3rd intervention session (T2, intervention arm only), post-intervention at 2 months (T3), and at 14 months (T4), i.e., 12 months after the intensive intervention to investigate maintenance of the changes immediately post-intervention (T3) (see Fig. 2). In vocational schools, 14 months is the most feasible follow-up period to optimise retention of students (before graduating or changing schools): a longer time-horizon would lead to higher drop-out rates due to difficulty in reaching the students. Measurements are conducted over six batches, corresponding to 6 study cohorts (Fig. 3), to even out burden of the research team. Thus, final follow-up data will be complete in May 2017. The trial employs methods to enhance quality of measurement (e.g., careful and repeated training of assessors including simulations of student contacts, exchanging assessors between schools in the same batches).

School staff usually estimate the number of students to be higher than it actually will be on the day of recruitment, meaning that not all students enlisted attend school. This is due to students being transferred to other classes, dropping out of school, or classes being scheduled for on-the-job learning period out of school, etc. Thus, if the students that are reachable have not attended the information session, the research group tries to reach them at school at least three times after this session. Despite this, not all students may be reached for information and consent forms.

#### Primary outcome measures

Objectively measured outcomes (MVPA, sedentary time, breaks in sedentary time) are based on accelerometry [32]. The 3-axial accelerometer (Hookie AM 20, Traxmeet Ltd, Espoo, Finland) has been shown to be a valid measurement tool both among adults [32] and young people [34]. The accelerometer is fixed to an elastic belt and participants are instructed to wear the belt on their hip for seven consecutive days during waking hours, except during shower and other water activities, starting from the delivery day. The accelerometers collected and stored the tri-axial data in raw mode in actual acceleration (g-units). The data is analysed in 6 seconds’ epoch length. PA was categorised into three intensity categories based on metabolic equivalents (MET): light, moderate and vigorous. Light PA is defined as activity corresponding 1.5−2.9 MET, moderate activity as 3.0–5.9 MET and vigorous activity more than 6 MET [35]. According to the definition of SB [36], time spent in sitting and reclining positions are combined to indicate SB, time standing still is analysed separately. It is possible to accurately determine whether the participant is standing, sitting or lying by applying the information from the raw data of the three measurement axes of the accelerometer. While the body position during walking is upright and the direction of Earth’s gravity vector is constant, the vertical position (angle) of the accelerometer can be identified during normal walking. This known position can then be used to recognise different body postures. In standardised conditions, standing can be separated from sitting or lying with 100 % accuracy [37]. Breaks in sedentary time are calculated on the basis of the number of lying/sitting periods ending-up with a clear vertical acceleration.

Research assistants deliver the accelerometers to the participants in schools, and they collect them the subsequent week at the school. All participants are instructed not to change their usual or intended patterns of activity when wearing the accelerometer. Self-reported PA during leisure time will be assessed using the validated NordPAQ measure [38] in the questionnaire, concerning MVPA over the previous seven days. The assessments take place concurrently in both intervention and control arms to control for the effect of weather conditions on activity. The assessors are not involved in intervening on students.

#### Other measures and materials

Table 3 shows an overview of measures among student participants with literature references. Secondary outcomes include self-reported SB, body composition, self-reported well-being, psychological variables (e. g. intention to restrict one’s SB), as well as health behaviours. Process measures include indicators of the direct behavioural predictors, i.e., the hypothesised mediators, such as PA intention, motivational regulations (self-determination theory), self-efficacy, and the use of BCTs (adapted based on an earlier study [39] and the Let’s Move It feasibility trial [20]). At T1 and T4, body composition is measured using Tanita MC-780MA bioimpedance measurement device. The questionnaire data is collected online (SurveyMonkey). Participating students complete the questionnaires in the school during a class session at T1-T4.

**Table 3:** Summary of measures among student participants in the Let’s Move It trial

	T1	T2^a^	T3	T4
Background information, covariates	X		X	X
PA & SB behavior				
Objectively measured PA & SB *(Hookie accelerometry)*	X		X	X
Self-reported PA, SB, and interruptions in SB [38, 42–46]	X	X	X	X
Other health related outcomes/covariates				
Objectively measured body composition *(Tanita MC-780 MA)*	X			X
Self-reported health & physical fitness [47]	X		X	X
Somatic symptoms (e.g. support- and mobility organ symptoms), stress, sleep [47] (e.g. [48–50]), dietary habits [51, 52]	X		X	X
Smoking	X			X
Psychosocial correlates of PA				
Behavioural beliefs: PA outcome expectations, PA descriptive norms (peers & parents), PA injunctive norm (parents), PA intention, PA self-efficacy/perceived behavioural control [53–55]	X	X	X	X
PA autonomous and controlled motivation [56]; integrated regulation subscale [57]	X	X	X	X
Opportunities for PA (school, neighborhood, at home)	X		X	X
PA automaticity [58]			X	X
PA action and coping planning [59]	X		X	X
Big five personality traits, brief measure [60]	X			
Psychosocial correlates of restricting SB^c^				
Behavioural beliefs: SB restriction outcome expectations, descriptive (peers) & injunctive norms (peers, teachers), self-efficacy/perceived behavioural control, intention [53–55]	X	X	X	X
Automaticity [58]			X	X
Autonomous and controlled motivation [56]			X	X
Perceived teacher behavior and group climate				
Perceived teacher actions to reduce students’ sitting, Perceived opportunities for SB reduction within school^b^	X	X	X	X
Teacher sitting reduction behavior (in school classes, perceived)^b^			X	X
Teacher motivational behavior for reducing student SB (e.g. discussions on SB at home/work practice)^b^			X	X
Student group climate (acceptance & safety) [61]	X	X	X	X
Behaviour Change Technique (BCT) use (enactment fidelity items)				
PA related BCT use: Frequency-dependent BCTs, Other BCTs ^b^	X	X	X	X
SB related BCT use^b^			X	X
Intervention receipt and evaluation of programme and materials^a^				
Recalled number of intervention sessions attended^a^			X	
Intervention satisfaction^a^			X	
Evaluation of intervention provider^a^ (autonomy support) [62]		X	X	
Evaluation and use of home workout videos, workbook & website^a b^			X	
Open-ended questions on intervention^a b^		X	X	
Adverse events				
Injuries or illnesses that prevent or limit PA	X	X	X	X
Perceived harmful effects from the intervention, open-ended^a b^			X	
Injuries				X

T1 = Baseline, T2 = Mid-intervention (after 3^rd^ intervention session), T3 = Post-intervention, T4 = Follow-up (14 months); PA = physical activity, SB = sedentary behaviour

^a^ = measured only of the intervention arm participants

^b^ = Questionnaire measure developed for this study

^c^ = existing measures adapted for this target behaviour

Teacher participants fill in questionnaires at T1, T3 and T4 after students’ measurements. Questionnaires measure behaviour (use of sitting reduction strategies), psychosocial determinants and use of BCTs, perceived social support from colleagues and supervisors, and background factors. Intervention arm teachers additionally fill in short feedback questionnaires after teacher workshops.

#### Harms and adverse events

PA-related injury and other perceived unintended negative consequences are self-reported by students at T3-T4. Adverse events are also be monitored constantly by project coordinators and intervention providers, and addressed in qualitative evaluation. Prior to the start of the trial, possible scenarios of adverse events were discussed (e.g., increased incidence of sports-related injuries, body dissatisfaction leading to eating disorders or steroid use for gaining muscle mass), and the team resolved to address these potential emerging effects promptly. However, the intervention content itself aimed to prevent such harmful effects by providing exercises to improve motor skills as a means for injury prevention, and by several exercises and discussions to prevent emergence of body dissatisfaction.

#### Economic evaluation

The economic evaluation will model the potential cost-effectiveness of the intervention in increasing PA and SB. Resources relating to intervention implementation will be collected and then used to calculate the costs relating to the added value of intervention with regard to primary outcomes. As the target group is not a patient population, health-related quality-of-life questionnaires are not used nor the longer term impact on quality-adjusted life years. Cost-effectiveness will be presented as the cost per added PA minute and reduced SB minute.

#### Qualitative process evaluation

Thematic, semi-structured individual interviews are conducted among a subsample of students, to evaluate e.g. receipt of the program and student interpretations of the causal mechanisms of change. A subsample of students from the control arm are interviewed to be able to make accurate conclusions about the mechanisms and effects of the intervention. Focus group interviews are conducted among a subsample of teachers, to evaluate e.g. receipt of the teacher workshops and identify needs for improvements before possible nation-wide dissemination. All interviews are recorded and transcribed. Additionally, field notes are kept and recorded during project meetings with teachers and during visits to schools.

Data management details can be acquired from the first author.

#### Strategies to ensure maximum study participation

The standard operational procedures for research assistants collecting the data explicitly advise and script friendly and respectful treatment of the students. We also offer coffee, tea, lemonade and biscuits during the data collection. *Questionnaire data*: While the consenting students fill in the electronic questionnaires, the non-consenting students are offered an alternative task: a written essay based on a dietary self-assessment test (at T4, on PA). This essay is in line with the schools health education curriculum and ensures these students are also focused on a meaningful task during assessment in-class. *Accelerometry*: Accelerometers are delivered to students with instruction and motivation on in a carefully scripted manner (at T1, one-on-one; at T3 & T4, in small groups). To avoid forgetfulness and support participants' use of the accelerometer in the mornings and assure wear for as long as possible, SMS reminders are sent early (6.30 – 7 a.m., during the internal pilot slightly later) to participants who do not opt out from this offer. To ensure retention at T4 and adequate use of the accelerometer, a movie ticket voucher is offered to all those returning the accelerometer promptly. At T1, T3 and T4, we offer for those who are willing to receive the print-out for their use of accelerometer. *Bioimpedance*: Bioimpedance measurements are conducted by a same-sex research assistant. Feedback is offered for those interested. To guarantee correct comprehension of the print-outs (and to avoid potential unintentional side effects, e.g., body dissatisfaction), they are given to students in a one-on-one personal consultation (2–5 min) after T1. Feedback is given in an identical way in both arms.

### Statistical analyses

Effectiveness analyses will be conducted according to the intention-to-treat principle. Primary and secondary outcomes will be analysed using generalised linear mixed model (GLMM) that will allow to take into account correlations between observations. These methods also allow the incorporation of incomplete longitudinal data into the analysis. Additional sensitivity analysis using the “as treated” approach will be conducted in order to gauge efficacy. Process analyses will include e.g. multiple mediation models to test whether the impact of the intervention is explained by its effect on the hypothesised mediators. We will conduct the outcome analyses separately for gender and different levels of baseline activity. When allowed by time and funding resources, we will also conduct the analyses by educational track, parental SES, and baseline body composition. Quantitative analyses will be conducted using the R, SPSS and the Mplus statistical softwares.

## Discussion

The study will address central gaps in the evidence base. The RCT will test the effectiveness of a multi-level theory-based intervention to increase activity among adolescents. To date, only three RCTs in this domain have evaluated effects on PA over the long-term, beyond one-month post-intervention [9]. This trial will show whether adding a carefully designed, evidence- and theory-based program will add value to teaching as usual. The analysis of intervention and BCT uptake and target groups’ experiences provide both theoretically interesting insights and also practically useful ideas to optimise intervention activities before wider dissemination. Investigations of fidelity and its predictors will provide practical help in the later dissemination phase in targeting key determinants and activities critical for maintenance.

To our knowledge, this is the first school-based PA intervention specifically targeted at vocational schools and tested in a randomised trial. The intervention addresses both individual and environmental features and provide evidence regarding the causal mechanisms and implementation, and their relation to outcomes. This matched pair design improves comparability of the arms (balanced for the most important covariates, gender and educational tracks) and thus improves power. Contrary to prior RCTs in the area that used an individual-level design, our class-level cluster design acknowledges the potential effect of small group dynamics on receipt of intervention activities delivered at class level.

The phased approach in development, combined with participatory process with end-users, may minimise risks such as infeasibility with the setting and target group, failure in recruitment, and inefficacy in producing or detecting the desired changes in behaviour due to inappropriate BCTs or poor statistical power. After the project ends, the anonymised dataset will be made available to other researchers.

The project also produces a practical program to be utilised in the Finnish school context. In line with the MRC recommendation [40, 41], we aim to promote dissemination and translation of the program into routine practice after completion of the trial. Vocational school students would benefit from increases in PA in terms of improved work capacity and mental well-being [4]. Intervention protocols including comprehensive manuals will be made available in the public domain, as well as the student workbook and other materials. The program components have the potential to be translated to be a part of the standard curriculum: Vocational school teachers, following appropriate training, can teach the student programme, the dose is easily embeddable in the school practices, and the student group program is compatible with the national curriculum learning objectives for health education. Thus, this intervention has high potential for dissemination. In order for a successful implementation into practice, the adoption should be supported by a training programme, consisting of a train-the-trainers manual, pre-intervention training, supervision and auditing organised by the research group, peer support and feedback systems at the schools, and on-line facilitator support materials.

### Ethical board

The Hospital District of Helsinki and Uusimaa, The Ethics Committee for gynaecology and obstetrics, pediatrics and psychiatry (decision number 367/13/03/03/2014.

### Trial status

Recruitment started in January 2015 and is ongoing.

## Additional file

Additional file 1: A selection of Let’s Move It posters, table triangle stands, and video workouts (screenshots). (DOCX 1.74 mb) Additional file 2: Accelerometer data by different cutoff criteria. (DOCX 20.0 kb)

## Abbreviations

BCT: behaviour change technique
BMI: body mass index
MVPA: moderate-to-vigorous physical activity
PA: physical activity
SB: sedentary behaviour
SES: socioeconomic status
