# Supplementary material for: ‘Let’s Move It’ – a school-based multilevel intervention to increase physical activity and reduce sedentary behaviour among older adolescents in vocational secondary schools: a study protocol for a cluster-randomised trial
Source: BMC Public Health. 2016 May 27;16:451. doi: 10.1186/s12889-016-3094-x (PMC4882860; doi:10.1186/s12889-016-3094-x)
Supplement: Additional file 1: — A selection of Let’s Move It posters, table triangle stands, and video workouts (screenshots). (DOCX 1.74 mb) [file 12889_2016_3094_MOESM1_ESM.docx]

Additional file 1: A selection of Let’s Move It posters, table triangle stands, and video workouts (screenshots).

Figure S1. Sample poster 1, benefits of physical activity.

Figure S2. Sample poster 2, increased energy levels as one of the benefits of physical activity.

Figure S3. Sample poster 3, ways of increasing activity into daily life.

Figure S4. Sample table triangle stand, sample stories of how to motivate oneself to be physically active.

Figure S5. Sample poster for teachers, ways of making classrooms active over the school year.

Figure S6. Sample poster for activity breaks: Brief exercises to increase relaxation.


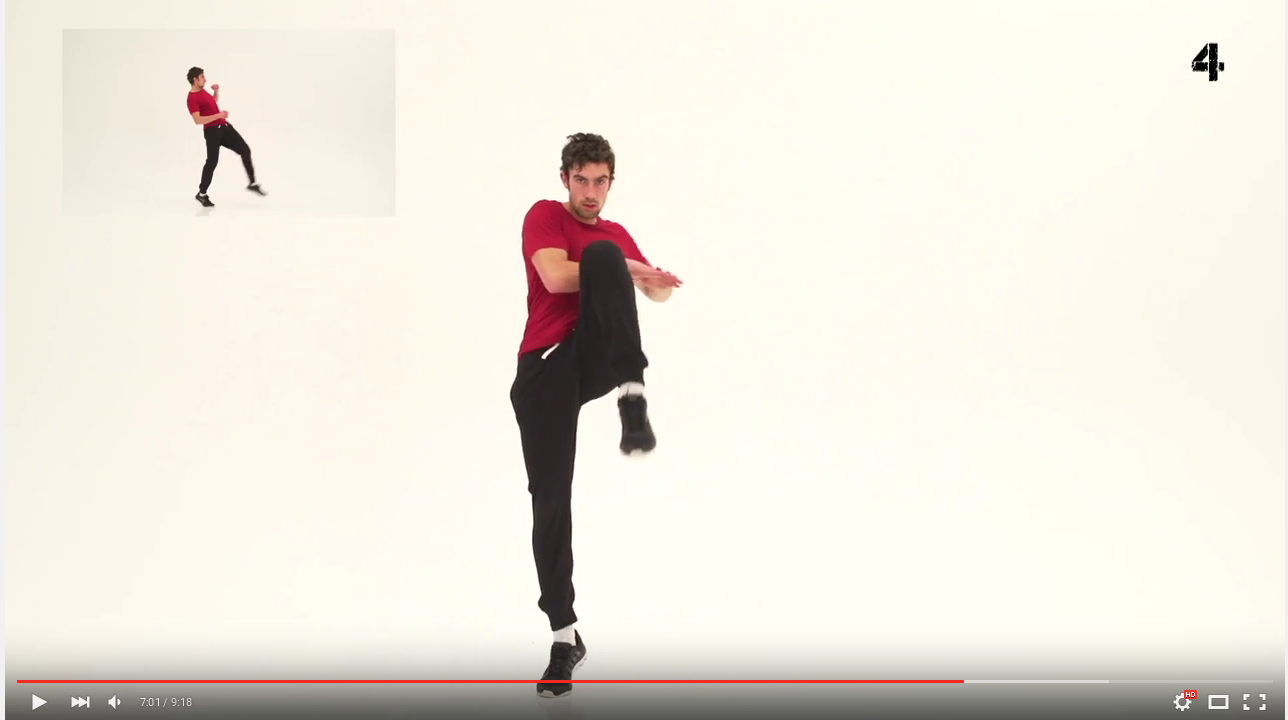


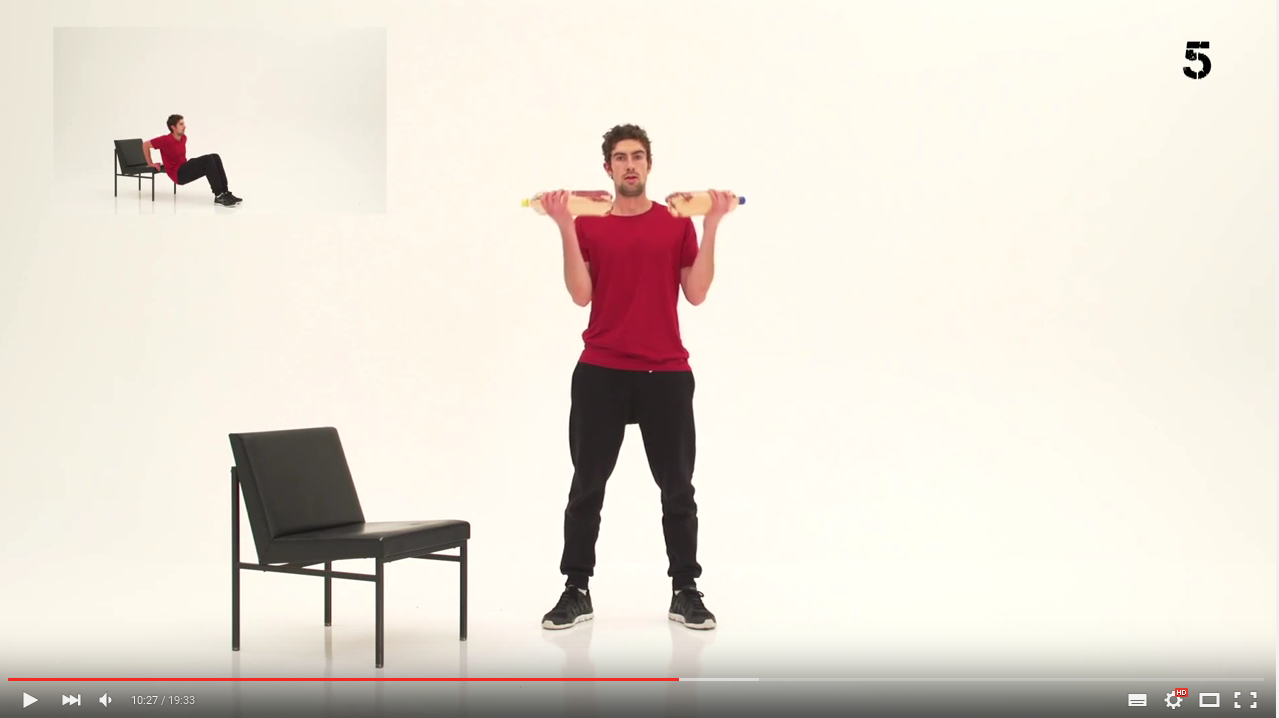


Figure S7. Screenshots of the Let’s Move It home workout videos for students.
