# Supplementary material for: ‘Let’s Move It’ – a school-based multilevel intervention to increase physical activity and reduce sedentary behaviour among older adolescents in vocational secondary schools: a study protocol for a cluster-randomised trial
Source: BMC Public Health. 2016 May 27;16:451. doi: 10.1186/s12889-016-3094-x (PMC4882860; doi:10.1186/s12889-016-3094-x)
Supplement: Additional file 2: — Accelerometer data by different cutoff criteria. (DOCX 20.0 kb) [file 12889_2016_3094_MOESM2_ESM.docx]

Additional file 2: Accelerometer data by different cutoff criteria.

Table S1. Accelerometer data by different cutoff criteria, internal pilot study (batches 1 & 2).

| Cutoff (days with > 10 hours of data) | Count | # of participants reaching the cutoff | % of participants reaching the cutoff |
| --- | --- | --- | --- |
| 0 | 34 | 376 | 100 % |
| 1 | 19 | 342 | 91 % |
| 2 | 24 | 323 | 86 % |
| 3 | 32 | 299 | 80 % |
| 4 | 39 | 267 | 71 % |
| 5 | 71 | 228 | 61 % |
| 6 | 67 | 157 | 42 % |
| 7 | 90 | 90 | 24 % |
